# Can health inequalities in sickle cell disease be addressed through novel therapies?

**DOI:** 10.1002/hem3.70175

**Published:** 2025-07-10

**Authors:** Stephen P. Hibbs, Richard J. Buka, Mary Shaniqua, Paul Greaves, John James, Paul Telfer

**Affiliations:** ^1^ Wolfson Institute of Population Health Queen Mary University of London London UK; ^2^ Institute of Cardiovascular Sciences University of Birmingham Birmingham UK; ^3^ Patient Advocate London UK; ^4^ Department of Haematology Barking, Havering and Redbridge Hospitals University Trust London UK; ^5^ Sickle Cell Society London UK; ^6^ Centre for Genomics and Child Health, Blizard Institute Queen Mary University of London London UK; ^7^ Department of Haematology Barts Health NHS Trust London UK

On 31st January 2025, the UK's National Institute for Health and Clinical Excellence (NICE) approved the gene therapy product exagamglogene autotemcel (exa‐cel) for use in some people living with sickle cell disease (SCD).^1^ This decision is a historical victory on several levels: a new potent treatment option for people living with a severe disease, an advocacy success to ensure access to a high‐cost medication for a marginalised group and a scientific milestone deploying CRISPR‐Cas9 gene editing in clinical practice.

Despite reasons for celebration, the reception to exa‐cel's approval is complicated. Although UK National Health Service (NHS) press statements and media reports focus on progress and hope, they rarely mention the recent history of two other novel therapies for SCD. These medications—crizanlizumab and voxelotor—were both developed specifically for people living with SCD, approved by NICE, administered to patients across the UK and withdrawn abruptly during 2024 due to efficacy and safety concerns^2^ (Figure 1).

**Figure 1:**
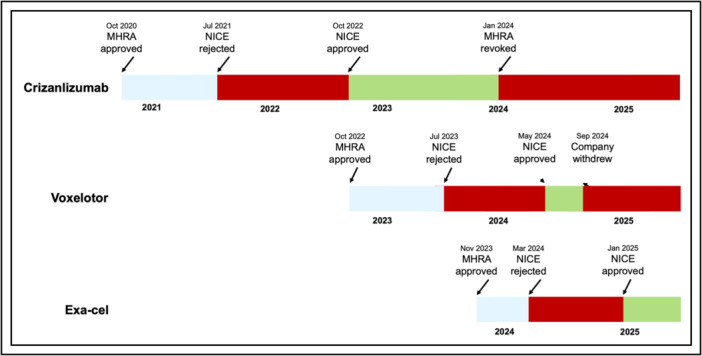
**Timeline of regulatory milestones for crizanlizumab, voxelotor and exagamglogene autotemcel (exa‐cel)**. MHRA, Medicines and Healthcare products Regulatory Agency; NICE, National Institute for Health and Care Excellence. Blue indicates the period following MHRA approval but before a decision by NICE. Green denotes availability through the National Health Service. Red denotes rejection by NICE or market withdrawal.

In this Perspective, we grapple with a challenging dilemma: Do health inequalities faced by people with SCD justify early access to new therapies? Or does reducing the requirement of clinical evidence expose patients to inadequately tested treatments, compounding inequalities as well as damaging trust? We argue that the current approach favouring early approval requires changes in how we communicate about regulatory decisions and how treatment withdrawals are navigated. Finally, we call for investment into ‘familiar therapies’: established but inadequately implemented strategies to address health inequalities for people living with SCD. Whilst our article is based on the UK experience, these considerations apply to novel therapies in diverse contexts.

## HEALTH INEQUALITIES AND TREATMENTS FOR SCD

Life expectancy and quality of life for individuals born with SCD have steadily improved over recent decades, but the condition continues to present profound challenges. Many patients suffer frequent hospitalisations and chronic organ damage, and some die prematurely from SCD complications.^3^ Most patients experience painful crises and significant fatigue. Although blood transfusions and hydroxycarbamide are accessible and transformative therapies for some, for others, these treatments are ineffective, unsuitable or cause intolerable side effects. Compounding the direct health impacts of SCD, individuals face significant health inequalities (Box 1). Patients, clinicians, advocates and regulatory bodies want to address these inequalities, in part through access to new SCD treatments.

Box 1 **Examples of health inequalities faced by people living with sickle cell disease (SCD)**

**Table:** 

– Most people living with SCD in Europe identify as Black African or Black Caribbean and are exposed to the effects of interpersonal and structural anti‐Black racism across many domains of life. – There has been inadequate investment in SCD research, which constrains clinical decision‐making and treatment options. Compared to cystic fibrosis—a genetic condition with a similar prevalence in the United Kingdom but primarily affecting White ethnic groups—SCD has received significantly less research funding, less research activity and fewer approved treatments.^4^ – Routine health services for people living with SCD were initiated only after decades of advocacy and remain inconsistent. – The 2021 All Party Parliamentary Group *No One's Listening* inquiry reported that SCD national care standards were poorly adhered to, healthcare professionals lacked awareness about SCD and patients frequently experienced negative interactions with staff.^5^

Three decades ago, a review article on SCD concluded that ‘the once‐distant goal of providing gene therapy for haemoglobinopathies is rapidly approaching reality’.^6^ In contrast to this prediction, the development of both gene therapy and other new disease‐modifying treatments has been frustratingly slow. In the two decades following hydroxycarbamide's adoption in the late 1990s, several new medications were trialled, but none were approved for SCD in the United Kingdom.

## THREE NEW DRUGS, THREE APPEALS AND TWO WITHDRAWALS

In the last 5 years, three novel therapies have been approved for use in the NHS for SCD: crizanlizumab, voxelotor and exa‐cel. The mechanism of action, clinical trial data assessed by NICE and excerpts from appraisals of the three therapies are summarised in Table 1. There are commonalities between their regulatory assessments: each was initially rejected by NICE on the grounds of insufficient clinical evidence and subsequently approved on appeal. In each approval on appeal, NICE argued that health inequalities faced by individuals with SCD were a reason to accept a greater uncertainty around clinical and cost effectiveness (Table 1).

**Table 1:** Key clinical trials and regulatory assessments for sickle cell disease novel therapies.

Therapy	Mechanism of action	Available trial evidence at time of NICE approval	Excerpt of NICE rationale for approval at appeal
Crizanlizumab	Monoclonal antibody which binds P‐selectin and inhibits adhesion between blood cells and vascular endothelium	SUSTAIN: a Phase 2 study of 192 patients. Patients randomised to high‐dose crizanlizumab had a 45% reduction in hospital admissions with painful crises compared to placebo.^7^	‘There is an unmet need for effective treatments for people with sickle cell disease. They also face health inequalities because the condition is not well understood, results in disability and is more common in people of African or African‐Caribbean family origin, who tend to have poorer health outcomes than other ethnicities. Access to crizanlizumab may help address these inequalities’^8^
Voxelotor	Small molecule which reduces polymerisation of intracellular haemoglobin S	HOPE: a Phase 2/3 study of 274 patients using a surrogate endpoint. 51% of patients randomised to higher dose voxelotor had at least a 1 g/dL increase in haemoglobin compared to 7% in the placebo arm.^9^	‘Greater uncertainty in the clinical‐effectiveness estimates could be accepted as a reasonable adjustment for the substantial disadvantages identified for people with sickle cell disease’^10^
Exagamglogene autotemcel (exa‐cel)	CRISPR‐Cas9 gene editing product which engineers haematological stem cells ex vivo at the enhancer sequence of BCL11A, resulting in increased haemoglobin F production	CLIMB‐SCD: a Phase 3 single‐arm study. At the time of publication, 30 patients had at least 12 months of follow‐up. 29/30 patients were free of vaso‐occlusive crises for 12 months.^11^	‘The committee took into account exa‐cel's potential impact on health inequalities (SCD is more common in people from African, Caribbean, Middle Eastern or South Asian family backgrounds)…by allowing more uncertainty in the evidence and a higher cost‐effectiveness estimate than NICE normally considers to be value for money for the NHS’^1^

Abbreviations: NHS, National Health Service; NICE, National Institute for Health and Clinical Excellence; SCD, sickle cell disease.

For crizanlizumab, NICE initially gave an unfavourable opinion because the SUSTAIN trial included a small number of patients and was short in duration. Following a successful appeal in 2021, partly because of health inequalities (Table 1), many SCD patients across the United Kingdom were treated with crizanlizumab under a managed access agreement. However, disappointment followed when the results of the Phase 3 STAND trial were presented in 2023, reporting no clinical benefit of crizanlizumab over placebo.^12^ In January 2024, crizanlizumab was withdrawn.

With voxelotor, an initial unfavourable opinion from NICE cited uncertainty about clinical effectiveness and high cost for the NHS. The drug manufacturer, Pfizer, and the Sickle Cell Society launched an appeal based on the UK Equality Act (2010), and NICE gave a positive recommendation in May 2024 (Table 1). For a brief period, voxelotor was approved and funded on the NHS. On 26th September 2024, within 4 months of the positive recommendation, Pfizer abruptly withdrew voxelotor, citing ‘an unfavourable imbalance in the number of vaso‐occlusive crises and fatal events in patients treated with [voxelotor]’.^13^ No data have been made publicly available.

The third novel therapy is exa‐cel. In March 2024, NICE issued provisional guidance that exa‐cel would not be recommended or funded for SCD because of insufficient evidence of benefit and inadequate cost‐effectiveness, while approving it for transfusion‐dependent thalassaemia. Two advocacy groups in the United Kingdom—the Sickle Cell Society and Anthony Nolan—sought responses to the provisional NICE guidance from SCD patients and clinicians. These charities asked respondents to emphasise the negative impacts of living with SCD, the worsening impact of the condition over time and the impact on life expectancy. Following this period of consultation, NICE issued updated guidance in January 2025, making exa‐cel available on the NHS through a managed access scheme,^1^ partly justified based on health inequalities (Table 1).

## THE DILEMMA OF UNCERTAINTY AND NOVEL THERAPIES IN SCD

The approvals and subsequent withdrawals of crizanlizumab and voxelotor have been deeply damaging. However, the celebration and promise of exa‐cel's approval represent the other side of the same coin. NICE appraisals of all three therapies have acknowledged that their normal threshold of clinical effectiveness was not met but that health inequalities justified lowering this requirement.

Herein lies a fundamental dilemma, arising in the context of historical underinvestment. Can lowering approval thresholds and allowing earlier access to novel therapies be a route towards health equity? Or does this approach remove vital protections and compound health inequalities for people living with SCD?

There are compelling arguments in favour of approving novel therapies in SCD based on limited evidence. Conducting clinical trials in SCD is challenging due to the lack of baseline mortality and morbidity data from existing clinical care, as well as difficulties in defining and measuring vaso‐occlusive crises within a trial setting.^14^ Furthermore, some trial participants have experienced their condition improving on these agents, and advocates within the community wish to see others benefit. Approving novel therapies can encourage further investment into SCD research and may attract some healthcare professionals to work within the field.

Conversely, there are strong arguments *against* lowering the required threshold of evidence to approve novel therapies. Individuals are at risk of harm from medications that have less evidence of benefit, especially when approval is based on surrogate endpoints, such as an increase in haemoglobin concentration for voxelotor. If therapies are subsequently withdrawn, this can have profound impacts on individuals: how does it feel to a patient to start crizanlizumab or voxelotor on the basis that it would ease the burden of SCD, and to experience a benefit, only to have the medication withdrawn months later and be told it is not safe or effective? The repercussions of medication withdrawal can disrupt multiple aspects of life. Furthermore, others within the SCD community observe flip‐flopping on decision‐making, compounding mistrust, suspicion and fatigue towards novel treatments and research. This may impact patient recruitment to future trials, exacerbating problems in gathering evidence.

Within our author group—a person living with SCD, an advocacy leader, clinicians and researchers in the SCD community—we hold different views on the level of evidence that should be required for approval of novel therapies. However, we agree that there must be clearer communication of regulatory approvals and a better response to any subsequent withdrawals.

## COMMUNICATING WITH CARE WHEN THERAPIES ARE APPROVED OR WITHDRAWN

Collectively, advocacy groups, clinicians and regulatory bodies have leaned towards earlier approvals of SCD novel medications, within a narrative of hope in new medicines as a route towards health equity. Considering this stance, we must learn from the examples of crizanlizumab and voxelotor about how to communicate transparently when novel therapies are approved or withdrawn.

When a new medication is approved, there is a temptation to celebrate unreservedly and overclaim on the significance of the approval. For example, the NHS England press release at the time of crizanlizumab's approval claimed that ‘This revolutionary treatment will allow patients to have a better quality of life, reduce trips to A&E by almost half and ultimately help to save lives’,^15^ despite the approval being based on limited Phase 2 data which did not assess mortality.^7^ Instead, public communication should cautiously celebrate while clarifying known toxicities, remaining uncertainties and the provisional nature of approvals. This honesty is also critical when seeking informed consent for individuals starting newly approved therapies.

We must better mitigate the damaging impact of future medication withdrawals if new data change the efficacy–safety balance of an approved drug. When medications are withdrawn, trialists and pharmaceutical companies must make data rapidly available to clinicians, researchers and patients so decision‐making can be understood. For SCD, this transparency has been lacking. Crizanlizumab was withdrawn based on results from the Phase 3 STAND trial, which was not published until March 2025—more than a year after its withdrawal.^16^ More than 6 months after Pfizer's press release announcing the withdrawal of voxelotor, we still have only a single paragraph of explanation and no available data to understand the decision.

Pharmaceutical companies must also invest in better support for individual patients if their medication is withdrawn. When voxelotor was withdrawn, clinicians and patients had no guidance on safe discontinuation, and no provision was made to support the mental health of affected individuals, in a condition where mental health challenges are already substantial.^17^ Some patients could not return to their previous treatments after novel therapy was withdrawn. If early approvals are to be granted to pharmaceutical companies, they must come with a requirement of *aftercare* for individuals affected by unexpected therapy withdrawals.

## ‘FAMILIAR THERAPIES’ AS ROUTES TO HEALTH EQUITY IN SCD

Finally, we must ask to what extent novel therapies can address health inequalities faced by people living with SCD. Currently, exa‐cel is the only remaining novel therapy available on the NHS. The NICE approval document estimates that 50 patients a year will be treated with exa‐cel.^1^ By comparison, around 17,000 people live with SCD in the United Kingdom. Most patients will not be eligible for exa‐cel, because they do not meet criteria such as age, genotype or severity. Some eligible individuals will decide against exa‐cel based on toxicities, including prolonged hospitalisation, infertility, secondary cancers and death. Globally, most patients with SCD live in low‐ and middle‐income countries, where there are no foreseeable prospects for exa‐cel being affordable or deliverable.

Routes towards health equity in SCD may come instead through investment in ‘familiar therapies’, which are known to be effective but are inadequately implemented. A recent study of NICE‐approved drugs (2000–2020) found that investing in existing services would have yielded greater population health benefits than funding new medications.^18^ One example is automated red cell exchange transfusion: a ‘familiar therapy’ which is effective at preventing many SCD complications,^19^ but for which eligible patients may wait for years before there is capacity for them to commence. Other examples include access to specialist psychology services^20^ and consistent emergency treatment of sickle cell crisis pain^21^—both recommended on national standards and guidelines, yet often unavailable to patients. Finally, it is crucial to address the barriers and toxicities of hydroxycarbamide, a highly effective ‘familiar therapy’ that improves survival.

## CONCLUSION

Individuals living with SCD face deep and broad health inequalities. Providing early access to new treatments creates opportunities for patient benefit whilst risking unknown harm and therapy withdrawal. We must communicate with greater care when novel therapies are approved or withdrawn. Finally, while engaging the potential of novel therapies to address health inequalities, healthcare organisations must also prioritise implementing ‘familiar therapies’ which remain inaccessible for many people living with SCD.

## AUTHOR CONTRIBUTIONS

**Stephen P. Hibbs:** Conceptualization; writing—original draft; writing—review and editing; project administration. **Richard J. Buka:** Visualization; conceptualization; writing—original draft; writing—review and editing. **Mary Shaniqua**: Conceptualization; writing—review and editing. **Paul Greaves**: Writing—review and editing. **John James**: Conceptualization; writing—review and editing. **Paul Telfer**: Supervision; conceptualization; writing—review and editing.

## CONFLICT OF INTEREST STATEMENT

S.P.H. and P.G. report no conflicts of interest. R.J.B. has received honoraria from SOBI, Bayer, Viatris and Sanofi, and research funding from AstraZeneca and SOBI. M.S. has participated in a paid sickle cell disease awareness campaign, funded by Pfizer. J.J. has not received personal payment from industry, but the Sickle Cell Society has received honoraria and grants from Novartis, Pfizer, Terumo BCT, Vertex and Novo Nordisk. P.T. has roles on advisory boards and committees for Pfizer, Vertex, Novo Nordisk and Agios, and research funding from Vertex.

## FUNDING

Stephen P. Hibbs is supported by a Health Advances in Underrepresented Populations and Diseases (HARP) Doctoral Research Fellowship 223500/Z/21/Z from the Wellcome Trust.

## Data Availability

Data sharing is not applicable to this article as no datasets were generated or analysed during the current study.
